# Calcium Imaging of Living Astrocytes in the Mouse Spinal Cord following Sensory Stimulation

**DOI:** 10.1155/2012/425818

**Published:** 2012-10-02

**Authors:** Giovanni Cirillo, Daniele De Luca, Michele Papa

**Affiliations:** Laboratorio di Morfologia delle Reti Neuronali, Dipartimento di Medicina Pubblica Clinica e Preventiva, Seconda Università di Napoli, 80100 Napoli, Italy

## Abstract

Astrocytic Ca^2+^ dynamics have been extensively studied in *ex vivo* models; however, the recent development of two-photon microscopy and astrocyte-specific labeling has allowed the study of Ca^2+^ signaling in living central nervous system. Ca^2+^ waves in astrocytes have been described in cultured cells and slice preparations, but evidence for astrocytic activation during sensory activity is lacking. There are currently few methods to image living spinal cord: breathing and heart-beating artifacts have impeded the widespread application of this technique. We here imaged the living spinal cord by two-photon microscopy in C57BL6/J mice. Through pressurized injection, we specifically loaded spinal astrocytes using the red fluorescent dye sulforhodamine 101 (SR101) and imaged astrocytic Ca^2+^ levels with Oregon-Green BAPTA-1 (OGB). Then, we studied astrocytic Ca^2+^ levels at rest and after right electrical hind paw stimulation. Sensory stimulation significantly increased astrocytic Ca^2+^ levels within the superficial dorsal horn of the spinal cord compared to rest. In conclusion, *in vivo* morphofunctional imaging of living astrocytes in spinal cord revealed that astrocytes actively participate to sensory stimulation.

## 1. Introduction

Two-photon laser scanning microscopy represents one of the main techniques for high-resolution *in vivo* imaging of central nervous system (CNS) [1–4]. By this approach have been revealed new details of neuroglial dynamic interactions in either physiological and pathological conditions [5–9]. This has generated a yield of knowledge causing the full revision of our understanding of neuroglial network plasticity. 

Astrocytes have been shown to play a key role in modulating neuroglial networks in the CNS [10–16] forming a structurally interconnected network functionally related to neurons. *Ex vivo* evidence has supported the astrocytic activation following neuronal activity, but it has never been established whether they respond to physiological activity *in vivo* [17]. 

Ca^2+^ oscillations occur spontaneously or in response to mechanical or electrical stimulation [18, 19]; few data have analyzed the astrocytic Ca^2+^ behavior during and after sensory processing. Cortical astrocytes have been extensively investigated [20, 21], but tools allowing imaging of glial cells in spinal cord *in vivo* are just emerging [22]. 

In this work, we have performed two-photon *in vivo* functional imaging of astrocytic Ca^2+^ dynamics in spinal cord. Pressurized injection of fluorescent dyes in the spinal cord specifically labeled astrocytes (red fluorescent sulforhodamine 101-SR101) and allowed monitoring of cytosolic Ca^2+^ dynamics (Oregon-Green BAPTA-1-OGB) during rest and after right hind paw stimulation. We found that Ca^2+^ levels in astrocytes significantly increase during sensory stimulation compared to resting. In conclusion, we directly imaged living astrocytes in spinal cord showing that astrocytes actively participate to sensory stimulation.

## 2. Materials and Methods

### 2.1. Animals

Adult (25–35 g; Charles River, Italy) male C57BL6-J mice (*n* = 10) were used. Mice were maintained on a 12/12 h light/dark cycle and allowed free access to food and water. Animal care was in compliance with Italian (D. L. 116/92) and EC (O. J. of E. C. L358/1 18/12/86) regulations on the care of laboratory animals. All efforts were made to reduce animal numbers.

### 2.2. Spinal Cord Window Surgery

**Figure 1:**
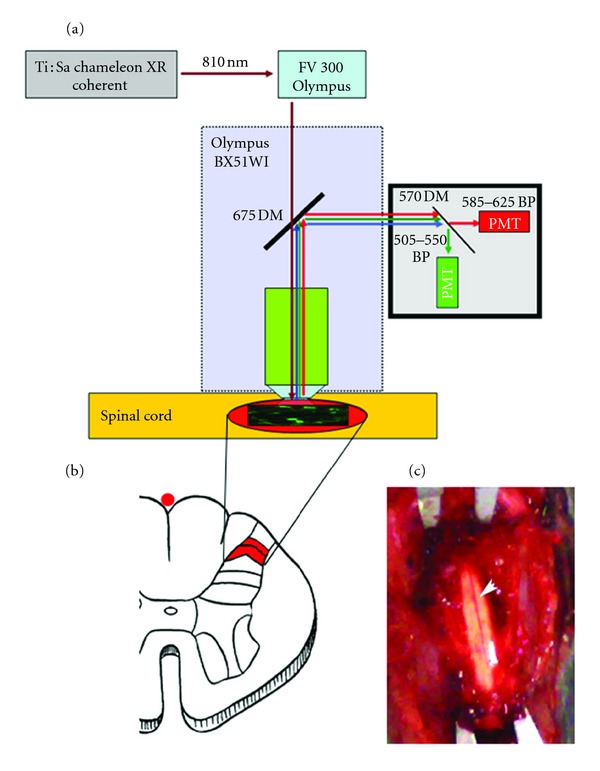
Experimental setup. (a) Two-photon laser scanning microscope setup. (b) Schematic section of the lumbar spinal cord showing the regions of interest (laminae I-II). (c) Exposed spinal cord surface, after vertebral laminectomy, before SR101/OGB loading. In the middle line, the posterior medial spinal vein.

Mice (*n* = 10) were anesthetized intramuscularly with 40 mg chlorohydrate tiletamine, 15 mg xylazine, and 2.5 mg acepromazine per kg in 0.9% NaCl solution. Anesthesia was then maintained using inhalatory sevoflurane (2% in 1 L/min oxygen). We minimized the breathing and heartbeat movements of the mouse using the Narishige device (STS-A, Narishige, UK). The skin of the back was incised and the muscles gently splayed to expose the thoracic vertebrae. We produced a custom spinal plate to be cemented on the vertebral surface, creating a well around the exposed vertebrae. The vertebral laminae were excised, and we exposed the lumbar spinal cord, leaving the intact dura [23, 24] (Figure 1(c)). The midline vein was visible through the vertebral window. We then filled the spinal well with a drop of artificial cerebral spinal fluid (ACSF).

### 2.3. Pressure Injection of Ca^2+^-Sensitive AM Dyes and SR101

Spinal astrocytes were loaded via pressurized micropipette injection (multicell bolus loading) *in vivo* with a Ca^2+^-sensitive dye under multiphoton microscope visual guidance. A patch pipette with a tip diameter of 2-3 *μ*m was inserted into the spinal cord to a depth of 150–300 *μ*m from the surface. OGB was dissolved in dimethyl sulfoxide (DMSO) containing 20% pluronic acid (F-127, Invitrogen) and mixed in artificial cerebrospinal fluid (ACSF) to a final concentration of 0.8–1.0 mM. A volume (10–15 *μ*L) of ACSF containing the dye was injected into the dorsal horn of spinal cord underneath the pial surface. The exposed spinal cord was also loaded with the astrocyte-specific indicator SR101 (Invitrogen, Italy). OGB and SR-101 were pressure-ejected from the pipette at 5–10 PSI for 60–90 seconds using the AM-system Picospritzer 2000. After injection, we allowed one hour for loading. To label vasculature, Dextran-Rhodamine (200 *μ*L of 20 mg/mL solution), which highlights blood plasma, was injected into the tail vein. 

### 2.4. Hind Paw Stimulation

Two subcutaneous copper needles were inserted into the right hind paw. Stimulus presentation was controlled by an analog-to-digital converter unit (AD instruments, UK) (pulse duration: 10 ms; amplitude: 1 mA; interpulse intervals: 167 ms/6 Hz). Three 30 s resting periods (R1, R2, R3) were allowed between two stimulations lasting for 10 s (S1, S2) [25].

### 2.5. *In Vivo* Imaging of the Mouse Spinal Cord: Image Processing and Quantification 

Throughout the imaging session (about 3-4 h from the beginning of surgery to the end of imaging), the anesthetized mouse was maintained with inhalatory sevoflurane at 37°C using a heating pad. Two-photon imaging of the spinal cord was performed using a custom-built two-photon laser scanning microscope (Figure 1(a)). It consists of a laser, a variable attenuator filter, a scanning unit, an upright microscope, and a photomultiplier (PMT-) based detection system. Laser is a tuneable titanium: sapphire (Chameleon XR; Coherent), whose wavelength range is from 690 nm to 1040 nm, power about 2.7 W, frequency 76 MHz. It was tuned at 810 nm to efficiently excite SR101 and OGB. To minimize photodamage, the excitation laser intensity was kept at a minimum for a sufficient signal-to-noise ratio (15–20 mW at the sample). The upright microscope is an Olympus BX51WI and is equipped with a water-immersion objective lens (Olympus) XLUMPlanFL 20XW, 0.95 NA. The scanning unit is a modified Olympus FV300. Fluorescence detection system has two channels that allows the simultaneous detection of two fluorescence signals. In order to efficiently collect SR101 and OGB signals, our system is equipped with a 570 nm dichroic mirror and bandpass barrier filters placed in front of the PMTs (505–550 nm for OGB channel and 585–675 nm for SR101 channel). Movement artifacts associated with the mouse heartbeat were overcome by triggering image acquisition from the mouse heartbeat (Powerlab, AD Instruments). The brightness and contrast of the acquired images were adjusted. To reduce the background noise associated with photon or photomultiplier tube noise, a median filter (radius, 1 pixel) was applied to each image. ImageJ free software was used (version 1.42, NIH, USA). 

### 2.6. Statistical Analysis

The fluorescent signals can be quantified by measuring the mean pixel intensities of the cell body of each astrocyte using ImageJ software. Movies were imported into ImageJ, and fluorescence traces were analyzed in spinal astrocytes and expressed as relative percentage changes (Δ*F*/*F*_0_) after background subtraction by exporting data to Sigma Plot 10.0 program (SPSS, Germany). We assumed that OGB-fluorescence intensity (Δ*F*/*F*_0_) directly correlated to intracellular Ca^2+^ levels [26]. Data are expressed as the mean ± SEM. Multigroup comparisons were made using an ANOVA with post hoc  *t*-tests. A *P* value ≤ 0.05 was considered statistically significant.

## 3. Results

### 3.1. *In Vivo* Imaging of SR101 Positive Astrocytes in Dorsal Horn of Lumbar Spinal Cord

**Figure 2:**
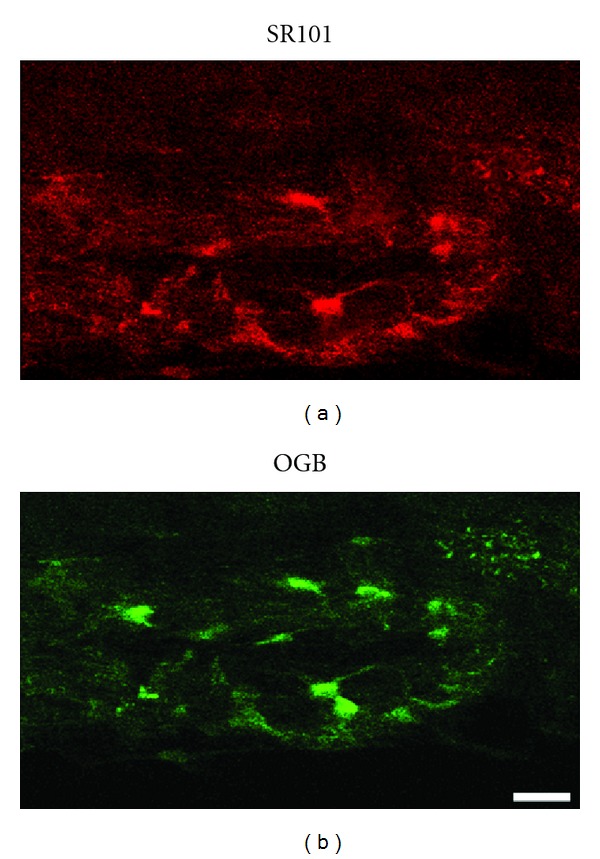
Selective labeling of astrocytes with SR101 and calcium indicator OGB. (a) A subset of SR101-labeled astrocytes in the superficial laminae of dorsal horn of lumbar spinal cord. (b) OGB-labeled astrocytes in the same ROI (scale bar: 20 *μ*m).

We used the posterior spinal vein, as a starting point (Figure 1(c)), and we follow the spinal blood vessels (Figure 4) to locate the dorsal horn of the spinal cord. Then, we imaged the area of posterior funiculus (Figure 1(b)): a depth of 200–300 *μ*m was sufficient to include the superficial laminae of dorsal horn of the spinal cord. Pressurized injection in the spinal cord of the red fluorescent dye SR101 resulted in rapid staining of astrocytes (Figure 2(a)) down to 300 *μ*m below the pial surface. All SR101-labeled cells showed morphological features of astrocytes. 

### 3.2. Ca^2+^ ****Levels of Spinal Astrocytes at Rest and following Right Hind Paw Stimulation 

Ca^2+^ oscillations of OGB-labeled spinal astrocytes (Figure 2(b)) were imaged during resting periods and following right hind paw electrical stimulation according to previously described stimulating protocol.

**Figure 3:**
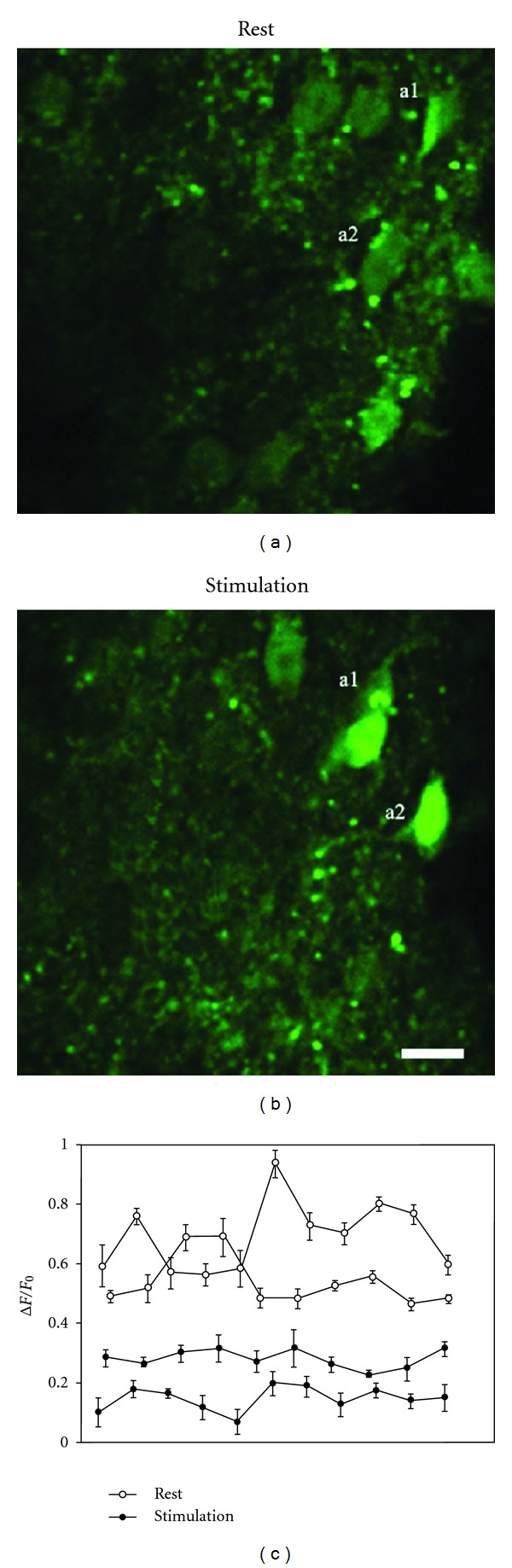
Sensory stimulation triggers Ca^2+^ increase in spinal astrocytes. Section of superficial laminae of dorsal horn of lumbar spinal cord, showing two different astrocytes (a1, a2) in rest condition (a) and following sensory stimulation (b) (scale bar: 10 *μ*m). (c) Quantitative analysis of astrocytic Ca^2+^ levels during rest and stimulation expressed as Δ*F*/*F*_0_. The mean value of Ca^2+^ increase during stimulation was significantly higher than that measured during rest (rest versus stimulus, **P ≤ 0.001).

**Figure 4:**
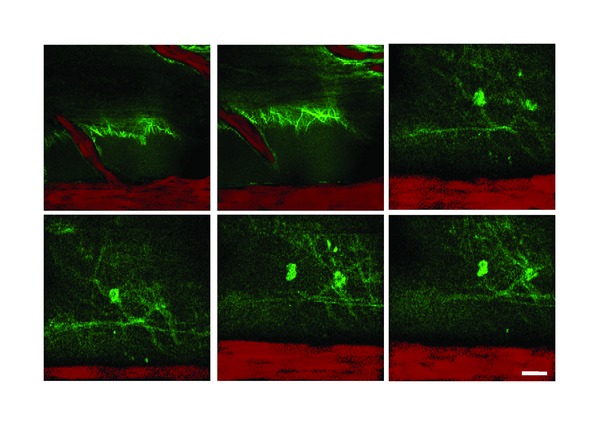
Time-lapse/*z* stack serial images of lumbar spinal cord. Spinal vessels are labeled with Dextran-Rhodamine; astrocytes adjacent to spinal vessels are labeled with OGB (scale bar: 10 *μ*m).

At rest, in anesthetized mice under normal conditions, astrocytes exhibited transient Ca^2+^ oscillations and this pattern did not significantly change during sensory stimulation. In contrast, right hind paw stimulation triggered increase of astrocytic Ca^2+^ levels (0.52 ± 0.03) (Figures 3(b) and 3(c)) compared to resting values (0.38 ± 0.02) (Figures 3(a)–3(c)). 

## 4. Discussion 

This study provides evidence that in the living spinal cord astrocytes respond to peripheral sensory stimulation with an increase of astrocytic Ca^2+^ levels. Time-lapse *in vivo* two-photon imaging of living spinal astrocytes, through intraspinal pressurized injection of dextran, SR101, and OGB, allows morphometric analysis of spinal microvasculature and spinal astrocytes, but also functional analysis of Ca^2+^ behavior. 

For decades, cell cultures and *ex vivo* analysis have greatly supported the study of CNS allowing the characterization of morphofunctional properties of neurons and glial cells. Although these studies have undoubtedly contributed to better understand the neuroglial biology, they have not pointed out the relevance of vascular but also cellular dynamic changes, of neuroglial mutual interactions, and properties of the living CNS. Along to this point of view, the development of *in vivo* imaging techniques has boosted the study of synaptic plasticity and functional properties of neuroglial networks. Moreover, the widespread application of technical facilities (imaging, surgical and stabilization techniques) has improved *in vivo* image acquisition ensuring an outstanding stability and repeatability of raw data. 

As recently reported [23], the use of a spinal stabilization device reduces artifacts caused by respiratory movements, improves the spinal column's stability, and allows direct data acquisition. Therefore, this technique allows a detailed study of neuroglial network of deeper layers in the spinal cord and can thereby provide functional data of cellular structures, intercellular interactions, and their changes over time. 

SR101-specific labeling of spinal astroglia permits the analysis of density and distribution of astrocytes *in vivo* and their role in neurodegenerative disease [27, 28] or following spinal cord or nerve injury [11, 12, 29, 30]. Colabeling other spinal elements, such as blood vessels and neurons, enables morphological and functional study of gliosvascular plasticity and neuroglial network [31]. Time-lapse imaging of astroglial end-feet and synaptic structures may help to elucidate their contribution to synapse formation and plasticity at the tripartite synapse [32].

We have presented a model to functional label astrocytes *in vivo* allowing morphofunctional imaging in the living spinal cord. OGB staining, in combination with SR101, enables the functional study of astrocytes *in vivo* and their role at the tripartite synapse in the spinal cord. 

As we recently demonstrated [27, 29, 31], glial response and the consequent adaptive synaptic plasticity lead to the concept of noncell autonomous disease and phenotypic changes affecting astrocytes play a key role in the onset and progression of the disease. Thereby, the availability of a reproducible and steady technique facilitates the widespread application of this powerful tool in studies of the spinal cord disease *in vivo*.

## Abbreviations

SR101: Sulforhodamine 101
OGB: Oregon-Green BAPTA-1
CNS: Central nervous system.
